# Advances in Clostridial Neurotoxins: Passage of the Intestinal Barrier and Targeting of Specific Neuronal Cells

**DOI:** 10.3390/toxins18010035

**Published:** 2026-01-10

**Authors:** Michel R. Popoff

**Affiliations:** Unité des Toxines Bactériennes, Institut Pasteur, Université Paris Cité, CNRS UMR 2001 INSERM U1306, 75015 Paris, France; popoff2m@gmail.com

**Keywords:** clostridial neurotoxins, botulinum neurotoxins, tetanus toxin, botulism, tetanus, motor neuron, central nervous system, receptor

## Abstract

Clostridial neurotoxins, botulinum neurotoxins (BoNTs), and tetanus neurotoxin (TeNT) are potent toxins responsible for severe diseases, botulism and tetanus, respectively. BoNTs associate with non-toxic proteins (non-toxic non-hemagglutinin, hemagglutinins, and OrfXs), which protect BoNTs against acidic pH and protease degradation and facilitate BoNT passage through the intestinal barrier. TeNT enters motor neurons and undergoes a retrograde axonal transport until the target inhibitory interneurons in the central nervous system. BoNTs and TeNT recognize specific cell surface receptors which consist of complex sets of protein(s)-glycan-gangliosides and determine specific cell entry pathways. Recent data on structural and functional investigations of BoNT and TeNT receptors bring a better understanding of toxin trafficking in the host and entry into target neuronal cells, which is useful for the development of updated strategies of prevention and treatment of the corresponding diseases. Since clostridial neurotoxins, notably BoNTs, are important therapeutic tools, detailed knowledge of their activity opens the way of the development of engineered molecules for specific clinical applications.

## 1. Introduction

Clostridial neurotoxins, including botulinum neurotoxins (BoNTs) and tetanus neurotoxin (TeNT), are among the most potent toxins and are responsible for severe neurological diseases in man and vertebrate animals, botulism and tetanus, respectively. A major common mechanism of BoNT and TeNT activity is the blockade of neuromediator release. While BoNTs prevent acetylcholine release from motor neurons at the neuromuscular junction leading to flaccid paralysis, TeNT blocks the release of glycine and GABA (gamma amino butyric acid) from inhibitory interneurons in the central nervous system (CNS), inducing spastic paralysis [1,2]. The high potency of clostridial neurotoxins results from cumulative effects of multiple steps of activity, including passage through the intestinal and endothelial barriers, targeting of specific neuronal cells, and interaction with critical components of the neuroexocytosis machinery [3]. Besides their drastic pathological effects, BoNTs are widespread therapeutic tools in an increasing number of clinical applications for humans. In recent decades, extensive works have been performed to define the structure and mechanism of action of clostridial neurotoxins to better understand the most potent toxins and to develop therapeutic tools with improved specificity and efficiency. More recently, the mode of BoNT passage through the intestinal barrier and the interaction of clostridial neurotoxins with neuronal cell surface receptors have been investigated in more details and are summarized in this review. Moreover, structural insights into the organization of BoNT complexes have been recently updated, allowing a more comprehensive understanding of the structure–function of these potent toxins. Following a reminder about the structure of clostridial neurotoxins and BoNT complexes, the aim of this short review is to synthesize the recent data on the following two major aspects of their activity: passage of the intestinal barrier and interaction with receptors on target neuronal cells with subsequent intracellular trafficking and activity shared by BoNTs and TeNT.

## 2. Structure of Clostridial Neurotoxins and Associated Proteins

According to their immunoreactivity regarding the neutralization of their paralytic activity by specific polyclonal antibodies, BoNTs are classified into seven toxinotypes (BoNT/A to BoNT/G). The most recently discovered toxinotype H is related to a hybrid between BoNT/F and BoNT/A [4,5,6]. Based on amino acid sequences, each toxinotype is subdivided into subtypes (more than forty) [7,8]. In contrast, TeNT shows limited diversity and only a single type is described [9,10].

BoNTs and TeNT share a common structure. They are synthesized as an about 150 kDa protein precursor which is proteolytically activated in a light chain (LC, about 50 kDa) and a heavy chain (HC, about 100 kDa), which remain linked by a disulfide bridge.

**Figure 1:**
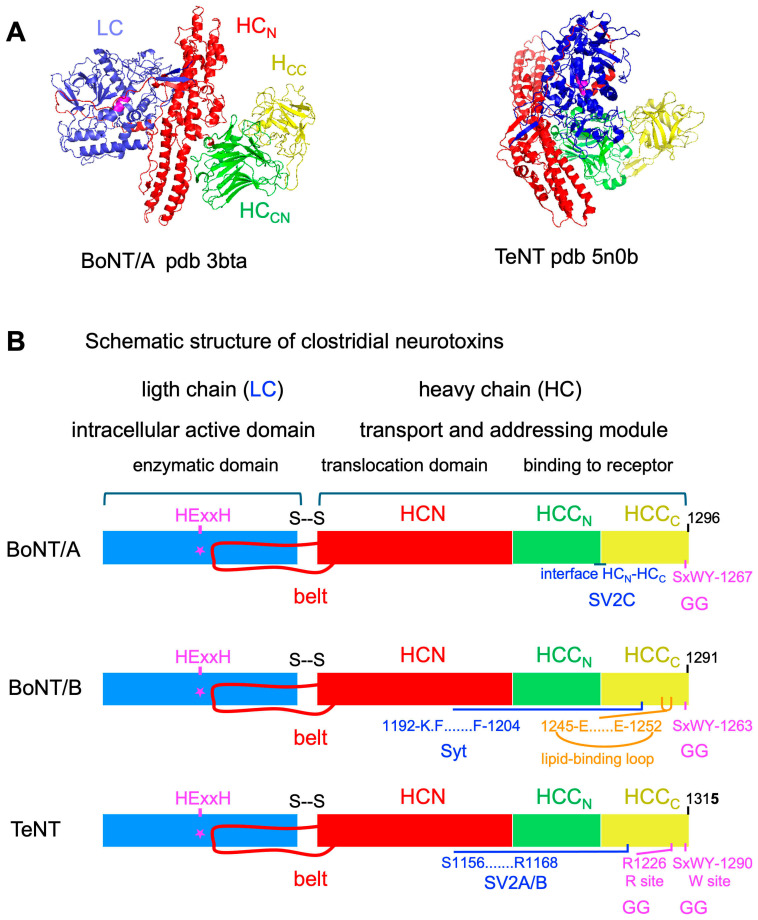
Structure of botulinum neurotoxin (BoNT) and tetanus neurotoxin (TeNT). (**A**) Comparison of the structure of BoNT/A and TeNT. The light chain (LC, blue) and the C-terminal parts of heavy chain (HCC_N_, green and HCC_C_, yellow) expand on each side of the N-terminal part of the heavy chain (HCN, red) in BoNT/A and on the same side of HCN in TeNT. (**B**) Schematic structures of clostridial neurotoxins indicating the enzymatic site (HExxH) in L chain and the binding sites to the receptors are as follows: gangliosides (GGs), synaptic vesicle protein 2 (SV2), and synaptotagmin (Syt) in HCC_C_ domain. Figures were produced with the program MacPymol v1.7.4.1.

LC contains the catalytic zinc-binding motif (HExxH) and is the intracellularly active domain. HC is the transport and addressing module of clostridial neurotoxins. HC consists of two main domains, HCN and HCC. HCN forms two unusually long and twisted α-helices and an extension, called the belt, surrounding LC. HCN is involved in the translocation of LC through the vesicular membrane. HCC is subdivided into two distinct subdomains, HCC_N_ and HCC_C_, which contain the receptor-binding sites. While the three structural and functional domains (catalytic, translocation, and receptor-binding domains) are linearly organized in most BoNTs, both the catalytic and receptor-binding domains are on the same side of the translocation domain in BoNT/E and TeNT. This domain organization in BoNT/E might facilitate a rapid translocation process of LC into the cytosol [1,2,11,12] (Figure 1).

**Figure 2:**
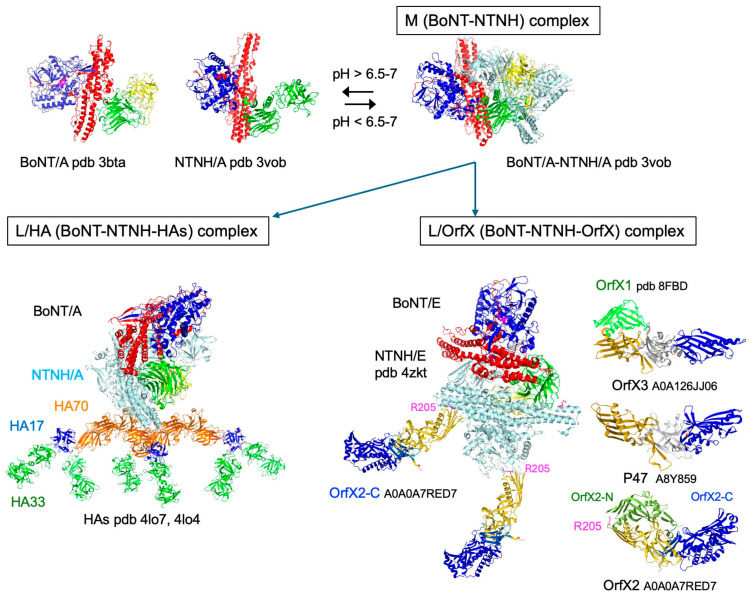
Structure of M complex resulting from the combination of BoNT with NTNH (non-toxic non-hemagglutinin protein), which shares a structure similar to that of BoNT, leading to a stable complex at low pH. M complex associates either with hemagglutinin components (HAs) forming a large dodecameric complex (L/HA) or with OrfX proteins (L/OrfX). Two OrfX2-C molecules bind to NTNH via R205, which has a major role, and OrfX1 associates with OrfX3. The organization of M-complex-OrfX2 with OrfX1/OrfX3 and P47 remains undetermined. Figures were produced with the MacPyMOL program.

BoNTs associate by non-covalent bonds with non-toxic proteins (ANTPs) produced by *C. botulinum* to form botulinum complexes of various sizes, also known as progenitor toxins. The medium-sized complex (M complex) consists of BoNT and the non-toxic non-hemagglutinin component (NTNH). Despite a low amino acid sequence identity, BoNTs and NTNHs share a similar structure, but NTNHs retain a modified non-functional catalytic site. In addition, the conserved ganglioside-binding site of BoNTs is not conserved in NTNHs. BoNT and NTNH form a tight interlocked complex at acidic pH which is resistant to protease degradation in acidic environment, while each component separately is rapidly degraded. M complex dissociates at neutral and alkaline pH [13,14] (Figure 2). Thereby, NTNH has a major role in the protection of BoNT in acidic environment such as in the digestive tract. NTNH also has a role in the appropriate activation of BoNT. The mode of BoNT activation has been elucidated in *C. botulinum* A from the group I *C. botulinum* strains which are proteolytic. α-Clostripain is the main protease which activates BoNT/A. α-Clostripain is synthesized as a pro-enzyme which is autoactivated in the external medium, thus avoiding BoNT/A degradation inside the bacteria. In the extrabacterial medium, BoNT/A associated with NTNH at low pH is specifically cleaved in LC and HC by α-clostripain. Thereby, NTNH protects BoNT/A from non-specific degradation by α-clostripain [15,16].

Large botulinum complexes (L complexes) result from the combination of M complex with either hemagglutinin components (HAs) or OrfX/p47 proteins. L/HA complexes are produced by certain *C. botulinum* A1 strains, *C. botulinum* A5, B, C, D, C/D, D/C, and G, while L/OrfX complexes are synthesized by certain *C. botulinum* A1 strains and *C. botulinum* A2, A3, A4, A5, A6, A7, A8, E, and F [17,18,19,20,21,22,23,24,25,26,27].

L/HA complexes contain three HA70 (70 kDa) molecules linked to NTNH. HA70 forms a trimer which assembles to NTNH via a loop (nLoop) in the N-terminal part of NTNH. Each HA70 binds to one HA17 (17 kDa) molecule which simultaneously interacts with two HA33 (33 kDa) molecules. Thereby, HAs assemble in a stable hetero-dodecamer complex at low pH through hydrophilic and hydrophobic interactions which adopt a triskelion-like fold (Figure 2) [21,28]. BoNT/A tightly associates with NTNH at pH 6.0–6.25 or less and dissociates at pH > 7.0 [14,29]. As monitored in BoNT complex from *Weissella oryzae*, the complex starts to dissociate at pH 6.5, it is about half-dissociated at pH 7.5, and completely disassembled at pH 8.5 [30].

**Figure 3:**
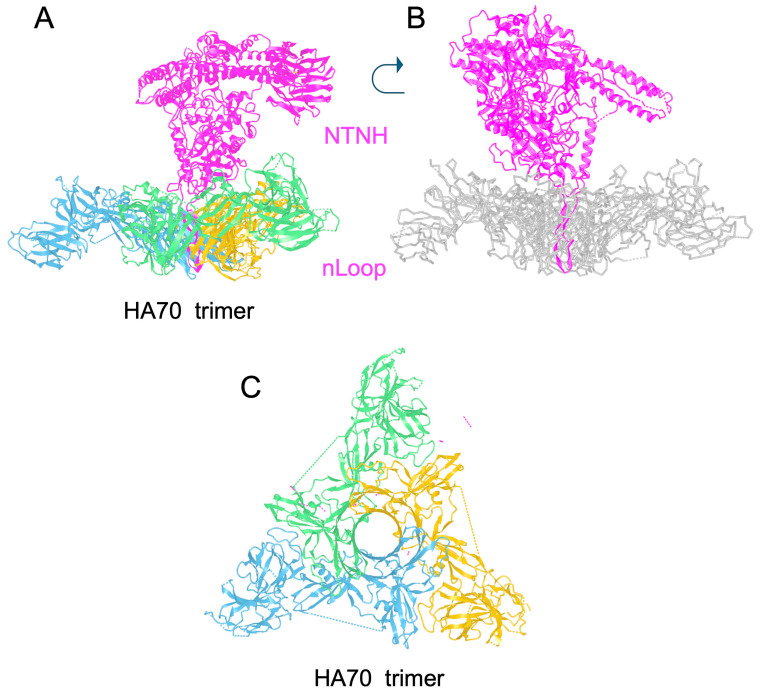
Assembly of NTNH with HA70 trimer from *C. botulinum* type B. (**A**) NTNH (magenta) and HA70 trimer (blue, yellow, and green). (**B**) NTNH and HA70 trimer (gray) highlighting the insertion of the nLoop into the center of the HA70 trimer. (**C**) Top of HA70 trimer showing the central pore. pdb 9QCO. Figures were produced with the CN3D program.

The assembly of BoNT/NTNH (M complex) with HA complex has been further investigated in BoNT type B. The long nLoop of NTNH/B (Gly111-Gly149), which is disordered in M complex, adopts a β-hairpin structure in L complex. The antiparallel β strands of the nLoop β-hairpin insert into the central pore formed by the assembly of three HA70s, leading to extensive contacts between the nLoop and HA70 (Figure 3). The assembly between NTNH nLoop and HA70 trimer, mainly mediated by electrostatic interactions between positively charged residues on the nLoop tip and negatively charged surface of the HA70 trimer pore, is highly stable. NTNH/BoNT dissociates from HA complex at pH 6.5–7.0, while BoNT starts to dissociate from its NTNH at lower pH (5.5–6.0). Interestingly, the nLoop is conserved in all NTNH types forming HA complexes and is lacking in NTNHs which associate with OrfX proteins [19,20].

L/OrfX complex consists of OrfX1 (17 kDa), OrfX2 (84 kDa), OrfX3 (55 kDa), and P47 (47 kDa) proteins, which are not organized in a compact complex, unlike L/HA complex. OrfX proteins share no significant identity with HAs. The main characteristic of OrfX and P47 proteins is their content in tubular lipid-binding (TULIP)-like domains. OrfX2 consists of two main domains, OrfX2-N and OrfX2-C. OrfX2-C, OrfX3, and P47 are elongated molecules containing three domains. The N- and C-terminal domains retain a TULIP-fold domain flanking a central β-sheet domain. OrfX2-N and OrfX1 consist of only one TULIP-like domain. OrfX1 interacts with OrfX3-N through polar and hydrophobic bonds. The OrfX1-OrfX3 complex shares a similar structure with that of OrfX2, in which OrfX1 mimics OrfX2-N and OrfX3 resembles OrfX2-C. While the two N-terminal TULIP-like domains of OrfX3 and OrfX2-C share a highly similar structure, their two C-terminal TULIP-like domains are more distantly related. Moreover, OrfX3 forms a homodimer through interaction between their C-terminal domains. Thus, OrfX3 linked to OrfX1 assembles in a hetero-tetrameric complex (Figure 2) [31,32,33]. Two OrfX2-C bind to NTNH via their N-terminal domains, leading to a BoNT-NTNH-OrfX2-C complex [34]. It is not yet clear whether OrfX3 and P47 associate with BoNT-NTNH-OrfX2-C to form a larger complex.

Interestingly, the *orfX-p47* cluster is widespread in non-clostridial species including Gram-positive and Gram-negative bacteria. In these bacteria, the *orfx-p47* cluster is located in the vicinity of genes encoding insecticidal toxins or other putative toxins, indicating that OrfX and p47 play a more general role than to specifically interact with BoNTs [35].

## 3. Botulinum Neurotoxin Passage Through the Intestinal Barrier

Botulism can be acquired from ingestion of BoNT preformed in food (food-borne botulism), intestinal colonization by *Clostridium botulinum* with BoNT production in the digestive tract (infant botulism, adult intestinal botulism), more rarely from wound contamination as in tetanus, toxin inhalation, and from cosmetic or therapeutic BoNT injection (iatrogenic botulism). In the majority of naturally acquired botulism cases, the initial and critical step is the BoNT passage through the intestinal barrier. Based on epidemiological investigations of botulism cases, the lethal dose of BoNT/A in humans is estimated to be 1 µg/kg by the oral route and 1–2 ng/kg by parenteral injection [36,37]. Thus, only a fraction of BoNT (about 0.1%) is transported through the intestinal epithelium, unless BoNT is partially degraded in the digestive tract. In experimental animals, the oral administration of the largest sizes of BoNT complexes correlates with the highest toxicity compared to pure BoNT (reviewed in [38]), supporting a protection role of the non-toxic associated proteins, notably NTNH, against digestive proteases [14], and possibly a role in the transport of BoNT through the intestinal epithelium. Furthermore, the toxin recovery rate in the lymph after intraduodenal administration of BoNT/A or BoNT/B complexes in rat was 0.01 to 0.1% [39,40]. Thus, BoNTs are poorly effective toxins to cross epithelial/endothelial barriers.

In the in vitro model of intestinal epithelial cells grown on filters, the passage of BoNT/A from apical to basolateral site is also very low (less than 1%). BoNT alone, without the help of ANTP, undergoes a receptor-mediated transcytosis through intestinal epithelial cells. Gangliosides GD1a/GD1b/GT1b seem to be the main BoNT/A and BoNT/B receptors on intestinal epithelial plasma membrane, without the requirement of a protein part receptor. As found for BoNT/A and BoNT/B, BoNTs enter intestinal cells via a non-clathrin-dependent pathway mediated by the small GTPase CDC42 and transit through non-acidified vesicles. Thereby, whole and fully active BoNTs are delivered through the basolateral side [41,42]. Investigations in mouse intestinal loop further support that the Hc domain of BoNT/A and BoNT/B drives the toxin passage through the intestinal epithelial barrier via a receptor-mediated transcytosis. Gangliosides of the GD1b/GT1b series, but not GM1, likely constitute the main BoNT receptors on intestinal mucosa. Only a subset of intestinal epithelial cells which still remain unidentified facilitate the toxin crossing through the intestinal barrier. In addition, BoNT/A likely transits through neuroendocrine intestinal crypt cells. In the intestinal mucosa, BoNT/A and BoNT/B target neuronal structures, preferentially cholinergic neurons, and to a lower extent other neurons such as serotonergic neurons and VIP (vasoactive intestinal peptide)-immunoreactive neurons [43,44,45].

L/HA complexes enhance the BoNT passage through the intestinal barrier by increasing the intestinal permeability. HAs bind to intestinal mucosa. HA70 and HA33 retain carbohydrate-binding sites with different specificities. HA70 preferentially recognizes sialyllactose and with lower affinity N-acetylneuraminic acid, while HA33 interacts with carbohydrates such as galactose, lactose, and acetyllactosamine [21,46,47]. The whole HA complex provides stronger binding activity than the individual HA components [46]. The multiple carbohydrate-binding sites of HA33 and HA70 mediate the HA complex to intestinal cell microvilli, whereas NTNH of type A do not [46,47]. However, NTNH from types C and D have been reported to bind to intestinal cells of human or rodent origin [48,49,50]. More recently, HA33 of type A strains such as A-62A was found to bind to fucosylated glycans, while HA33 of type B strain Okra, which is hyper-oral-toxic, did not. His281 in HA33 of A-62A is the major determinant for fucose binding and is replaced by an Asn in HA33 of B-Okra. Fucose is a major component of glycoproteins, such as those from mucin of the mucus layer which covers the intestinal epithelium. Thus, in contrast to type A-62A L/HA complex, which is trapped in the mucus layer, the type B-Okra L/HA complex has access to and enters epithelial intestinal cells, supporting its high oral toxicity [51,52]. Toxin crossing of the mucus layer is the first step in foodborne intoxication. Intestinal mucin fucosylation is mediated by enzyme FUT2, which adds an L-fucose to O-glycans. About 20% of persons carry a non-functional FUT2 (called “non-secretors”) and are more susceptible to certain intestinal disorders, such as Crohn’s disease, as well as more resistant to other intestinal pathogens (norovirus, rotavirus, *Helicobacter pylori*, *Vibrio cholerae*, and enterotoxigenic *Escherichia coli*) [51,53,54]. FUT2 polymorphism is possibly a susceptibility factor for foodborne botulism. “Secretor” persons could be more resistant to BoNT/A or B associated with A-62A L/HA type. Interestingly, *C. botulinum* B4, which produces HAs with the A-62A profile [51], is prevalent in human botulism after consumption of pork products, such as in France [55,56]. Pigs are frequent healthy carriers of *C. botulinum* B and rarely develop clinical botulism [57]. The high affinity binding of type B4 L/HA to pig gastric mucin [51] might contribute to pig healthy carriage. Interaction of type B4 L/HA with human mucin remains to be investigated.

**Figure 4:**
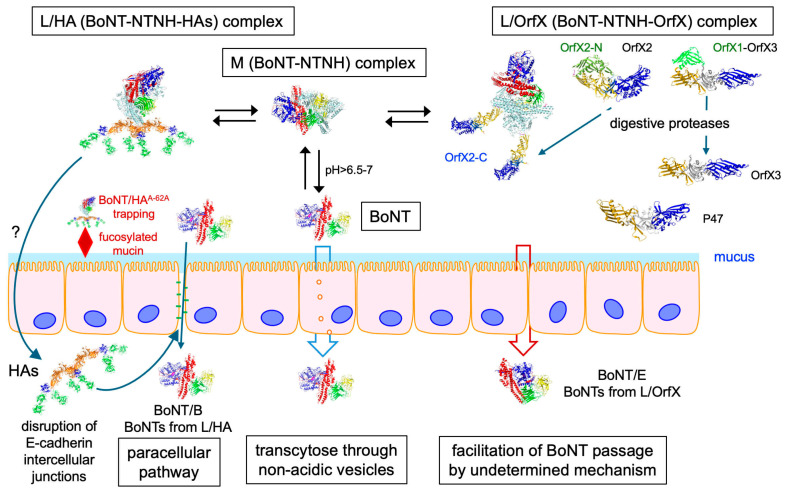
Schematic representation of the modes of BoNT passage through the intestinal barrier. BoNT alone can transcytose through intestinal cells, but with low efficiency. HA complex transcytoses through M cells or other undefined cells, binds to intestinal epithelial basolateral side, and disorganizes E-cadherin intercellular junctions, thus allowing BoNT passage via the paracellular way. OrfX proteins facilitate the intestinal absorption of BoNT through a yet undetermined mechanism. Mucin fucosylation traps BoNT/A or BoNT/B associated with type A-62A HAs in the mucus layer preventing access to intestinal cells.

Moreover, the whole HA complex, but not the dissociated HA components, binds to E-cadherin on the basolateral side of intestinal epithelial cells. The C-terminal part of HA70 and to a lower extent HA33 and HA17 have an essential role in E-cadherin binding. The E-cadherin binding sites on HA complex are distinct from those involved in acid sialic/carbohydrate interactions [58,59]. Binding of HA complex to E-cadherin leads to a disassembly of E-cadherin interactions in the adherens junctions as well as disorganization of the tight junctions which facilitate BoNT passage through the intestinal barrier by the paracellular pathway [21,59,60]. HA complexes from different *C. botulinum* types show distinct host specificity. Type A and type B HA complexes disrupt human, bovine, and mouse intestinal epithelium integrity, while type C HA complex does not interact with human E-cadherin but adheres to intestinal cells of susceptible animals and alters, for example, rat intestinal cell barrier. Type B HA complex is inactive on rat or chicken epithelial cells [61,62,63]. These different HA complex specificities are related, at least partially, to distinct HA33 binding features. Type C HA33 binds to lactose with an about seven-fold lower affinity than type B HA33 and interacts with N-acetylneuraminic acid, in contrast to types A and B HA33 [64]. The host specificity of the HA complexes from the different *C. botulinum* types accounts, at least in part, for the occurrence of botulism types in human and animal species. For example, botulism type A and B are prevalent in humans, while botulism type C and D are extremely rare [57]. How does HA complex migrate through the intestinal barrier to target E-cadherin on the basolateral side of intestinal epithelial cells? It has been proposed that HA complex translocates through the microfold (M) cells. Indeed, type A HA complex recognizes the glycoprotein 2 (GP2) on M cells [65]. However, BoNT is mainly absorbed from the upper small intestine [66,67], whereas M cells are mostly distributed in the distal part of the small intestine. Moreover, the passage of type A L/HA complex was similar through intestinal cell monolayers containing or not containing M-like cells [42]. Alternatively, HA complex might undergo transcytosis through intestinal epithelial cells [59,61] (Figure 4). It is noteworthy that most of BoNT is likely dissociated from ANTPs in the intestinal content since the pH is usually neutral or slightly alkaline, at least in long intestinal parts (pH in human small intestine 7.4–7.5 [68] or 6.4–8.2 [69], bovine pH > 6.5 [70], and bird pH 6.2–7.4 [71]). In contrast, the intestinal pH of species in which orally acquired botulism is extremely rare is lower (rat, mouse pH < 6.5, pig pH 6.1–6.7) [72,73]. Thus, the intestinal pH seems to be an important factor on the stability of BoNT complexes and subsequent BoNT absorption through the digestive barrier and should be further investigated.

More recently, the role of L/OrfX in the intestinal absorption of BoNT has been investigated. Oral toxicity of botulinum E complex lacking or containing mutated OrfX/P47 proteins was 40- to 90-fold lower compared to wild type botulinum E complex, while the toxicity by intraperitoneal route was similar with both wild type and altered botulinum E complexes. Thus, OrfX/P47 proteins show a specific role in the oral toxicity of BoNT by enhancing the toxin passage through the intestinal barrier. It is noteworthy that in the absence of functional OrfX/P47 proteins, sufficient amount of BoNT/E passed through the intestinal barrier and caused the death, supporting that BoNT is able by itself to cross the intestinal epithelium albeit with a low efficiency [34] (Figure 4).

OrfX1 and OrfX2-N are sensitive to protease digestion and are degraded in the intestinal content, while OrfX2-C, OrfX3, and P47 are resistant. OrfX1 and OrfX2-N are likely chaperone proteins involved in the protection of OrfX2-C and OrfX3, respectively. Upon degradation of OrfX2-N, two OrfX2-C molecules bind to NTNH, forming the complex BoNT/E-NTNH-OrfX2-C (Figure 2). It is not yet clear whether OrfX3 and P47 structurally assemble with M complex. The four OrfX/P47 proteins are required for full oral toxicity. The TULIP domains of OrfX2-C, OrfX3, and P47 which are known to interact with lipids might drive BoNT absorption through the intestinal barrier by a yet unidentified mechanism [34]. The related OrfXs/P47 proteins in non-BoNT-producing bacteria possibly form a similar machinery for intestinal absorption of toxins or other proteins [34,35].

## 4. Neuronal Cell Surface Receptors of Botulinum Neurotoxins

A hallmark of clostridial neurotoxins is their specific interaction with certain neuronal cell types. A double receptor model, including a ganglioside and a membrane protein, has been proposed for BoNT and TeNT neuronal receptors [74,75]. Ganglioside-binding site and protein-binding site are distinct and are localized on the HCC domain of the clostridial neurotoxins.

BoNTs bind preferentially to GT1b, GD1b, and GD1a, and TeNT to GT1b, GD1b, and GQ1b, with varying affinities. The ganglioside-binding site containing the core motif SxWY is conserved on the tip of the HCC_C_ domain of TeNT and BoNTs, except in BoNT/C and BoNT/D. TeNT shows an additional ganglioside-binding site with R1226 as key residue, which interacts with sialic acid (Figure 1) [1,76,77]. Thus, ganglioside binding confers a high TeNT affinity for neuronal membrane [78]. A lipid-binding loop is particularly developed between the ganglioside- and protein receptor-binding sites in BoNT/B, DC, and G. The lipid-binding loop contributes to toxin interaction with neuronal membrane in a ganglioside-independent manner, as shown in BoNT/DC [79,80].

**Figure 5:**
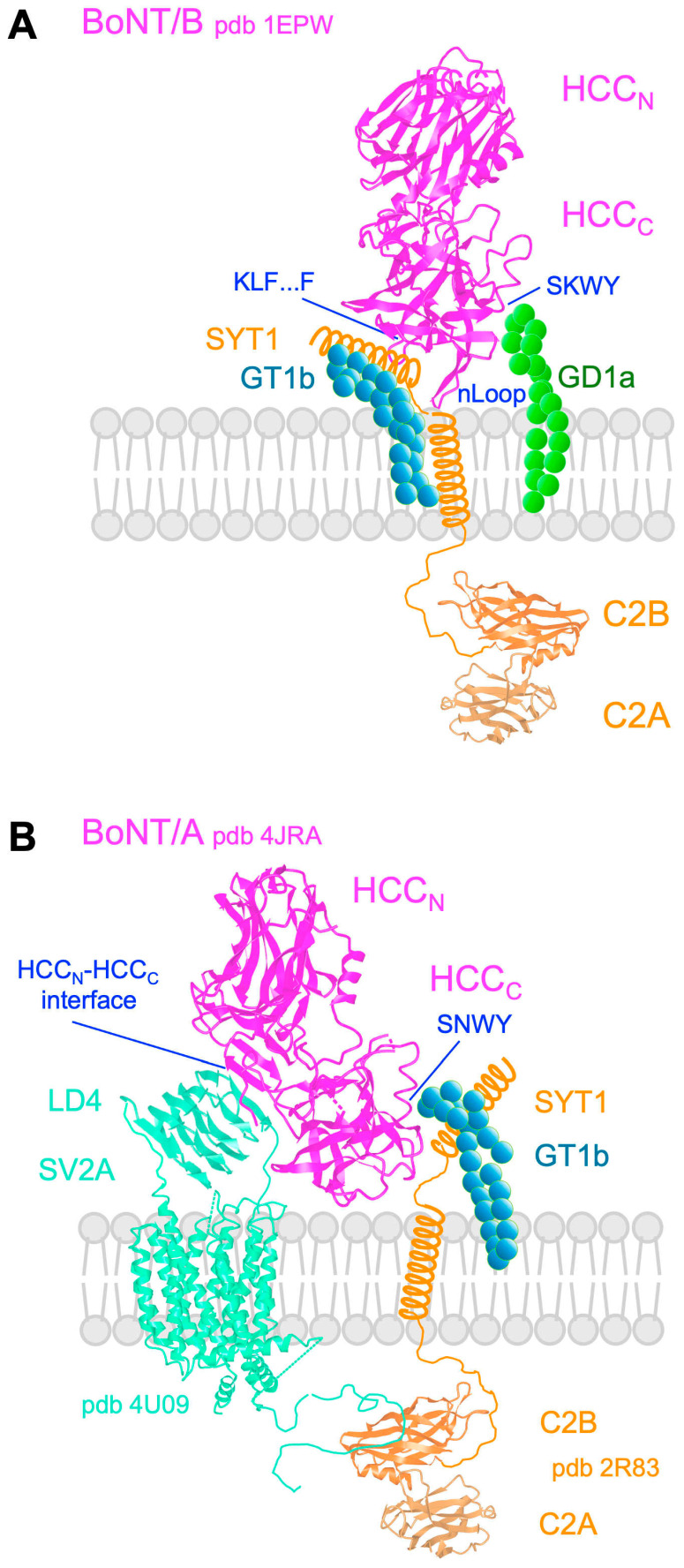
Interaction of BoNT with neuronal cell surface receptor(s). (**A**) BoNT/B binds to ganglioside GD1a through its ganglioside-binding site in the HCC_C_ domain and to synaptotagmin (Syt) preassembled with GT1b through the Syt binding site, adjacent to the ganglioside binding site in the HCC_C_ domain. (**B**) BoNT/A binds to ganglioside preassembled with Syt-I through the ganglioside-binding site on the tip of HCC_C_ domain and to the luminal domain LD4 of the vesicular synaptic protein 2 (SV2) via the binding site at the interface of HCC_N_ and HCC_C_. The figures were produced with the program CN3D.

The protein part of BoNT receptors includes the synaptic vesicle proteins 2 (SV2s) for BoNT/A, E and F, and synaptotagmin (Syt) for BoNT/B, DC, and G. Albeit SV2 is required for the entry of BoNT/D into neuronal cells, no direct interaction between BoNT/D with SV2 has been shown [1,77]. SV2 belongs to the transmembrane transporter family and is expressed in neuronal and endocrine cells. SV2 is a 73 kDa protein harboring twelve trans-membrane domains, six luminal loops, including a large luminal loop (domain 4 or LD4) with three N-glycosylation sites, and a long cytoplasmic N-terminal domain. Three isoforms, SV2A, SV2B, and SV2C, have been identified. SV2s are important regulators of the Ca^++^-dependent exocytosis. However, the multifunctions of SV2s are not yet clearly defined [81,82]. Syt is a 65 kDa protein anchored in the synaptic vesicle membrane which triggers the neuroexocytosis upon binding to Ca^++^. Syt contains a single membrane-spanning domain, a short luminal domain, and two cytoplasmic Ca^++^ sensing domains (C2 domains) (Figure 5) [83].

BoNT/A binds to the luminal domain 4 (LD4) of SV2A, SV2B, and with higher affinity to that of SV2C, whereas BoNT/E interacts with SV2A and SV2B, but not with SV2C. The binding site of SV2C-LD4, which forms a quadrilateral β-helix containing pentapeptide repeats, is located in the interface between HCC_N_ and HCC_C_ subdomains of BoNT/A [84,85,86] (Figure 1 and Figure 5). In contrast to BoNT/A, BoNT/E uses two distinct SV2A-LD4-binding sites, a protein–protein-binding site localized in HCC_C_, which is in a more closely related position than the Syt-binding site in BoNT/B, and a glycan-binding site in the HCC_N_ subdomain [87,88]. BoNT/E interacts with the side of the quadrilateral helical bundle of SV2A-LD4, while BoNT/A recognizes the open edge of the SV2C-LD4 structure [87].

Recognition of carbohydrate motifs plays a determinant role in the specific interaction of BoNTs with their neuronal receptors. Indeed, the glycan moiety determines the BoNT specificity to gangliosides. Notably, the terminal trisaccharide motif of ganglioside is essential for the specific interaction with BoNTs [89,90]. Thus, BoNTA1, A2, B, E, and F bind with high affinity to gangliosides containing these oligosaccharides linked to *N*-acetylneuraminic acid (GT1b, GD1a) and poorly to gangliosides lacking these terminal sugars (GD1b, GM1a) [89]. Moreover, carbohydrate recognition is also involved in BoNT interaction with its receptor protein part. In addition to peptide–peptide interaction, the recognition of glycosylated Asp559 (N559 glycan) in the luminal domain of SV2A, SV2B, and SV2C confers higher binding affinity to BoNT/A. The residues involved in glycan interaction are conserved in all BoNT/A subtypes except in BoNT/A4 (Arg at position 1292 instead of Gly), and thus BoNT/A4 retains a very low biological activity [91,92,93]. SV2 glycosylation plays also a crucial role in binding to BoNT/E, which only interacts with glycosylated SV2A and SV2B, but not with the unglycosylated isoforms [88,94]. Glycosylation at N573 in SV2A-LD4, which is equivalent to SV2C N559 glycan, is essential for BoNT/E entry into neuronal cells [94]. The SV2A-N573 glycan-binding site in the HCC_N_ subdomain of BoNT/E is distant from the protein–protein interaction site in the HCC_C_ subdomain, whereas in BoNT/A, both glycan- and protein-binding sites of SV2C are closely localized in the HCC_N_-HCC_C_ interface. The glycan recognition likely contributes to the discrimination of SV2 isoforms by BoNT/A and BoNT/E.

The binding site to Syt luminal domain, which is a short α-helix, has been mapped in the HCC_C_ domains of BoNT/B, DC, and G upstream of the lipid-binding site, which includes the segment K1192-F1204 in BoNT/B, and the ganglioside-binding site on the tip of HCC_C_ (Figure 1). BoNT/B and DC use Syt-II and to a lesser extent Syt-I as protein part receptor, whereas BoNT/G displays similar affinities to both Syt-I and Syt-II. The Syt-binding pocket of BoNT/DC is closely related to those of BoNT/B and G, but in a different orientation [77,95,96,97]. Sequence variation in the Syt luminal domain induces different species specificity of BoNT/B, DC, and G. In contrast to rat and mouse Syt-II, human and chimpanzee Syt-II shows a lower binding affinity for BoNT/B and BoNT/G due to a unique mutation in Syt-II luminal domain (Leu51 in human Syt-II instead of the corresponding Phe54 in mouse Syt-II) [98]. This partially accounts for the weaker type B botulism symptoms and lower efficiency of therapeutic BoNT/B in humans compared to BoNT/A. Furthermore, BoNT/DC retains the highest affinity for bovine Syt-II and no detectable binding to human Syt-II, in agreement with the prevalence of botulism DC in cattle, whereas this type of botulism has not been reported in humans [99]. Syt-I and Syt-II are homologous proteins, but with different distribution in neuron types [100]. Syt-I is widely distributed in brain neurons, notably in forebrain, and in autonomic and sensory neuron endings, whereas Syt-II is dominant in brain caudal areas and in peripheral motoneurons [101,102,103]. Based on knockin mouse mutants, Syt-I is the major BoNT/B receptor on autonomic neurons controlling smooth muscles such as those in bladder, and Syt-II is predominant at the skeletal neuromuscular junctions [104]. This correlates with human botulism type B, which is characterized by a predominance of dysautonomic symptoms [105,106].

In contrast to BoNT/A, glycosylation is not required for BoNT/B interaction with the Syt luminal domain. Gangliosides, preferentially GT1b and GD1a, enhance BoNT/B binding to Syt-I and Syt-II, allowing the toxin entry into neuronal cells. Moreover, the function of Syt-I as BoNT/B receptor is strictly dependent on gangliosides [74,75,76,77]. Indeed, BoNT/B binding to Syt-I or GT1b alone in membrane context was observed, but only when both Syt-I and GT1b are included [107,108]. Thus, high BoNT/B affinity receptor consists of Syt-I or Syt-II associated with gangliosides. A glycosphingolipid-binding site has been identified in the extracellular juxtamembrane domain of Syt-I and Syt-II, which overlaps for the most part with the BoNT/B binding site. GT1b interacts with the glycosphingolipid-binding domain of Syt-I and Syt-II, with a similar affinity constant through hydrophobic residues. Thereby, preassembled GT1b-Syt constitutes the high affinity receptor recognized by BoNT/B [108,109]. The main effect of GT1b binding to Syt is the transition of the Syt extracellular domain from a random structure to an α-helix, which is required for BoNT/B binding to Syt. Additionally, GT1b might induce an electrostatic attraction and stabilization of the receptor [108]. As glycans are important in BoNT neuronal specificity, ganglioside glycan might compensate the absence of glycan in the Syt luminal domain. As mentioned above, BoNT/B contains two receptor-binding sites (GT1b and Syt binding sites), closely located but not overlapping on the toxin HCC tip, thus enabling their concomitant interaction with their corresponding residues on BoNT/B receptor, which is constituted of a unique ganglioside–Syt complex. A lipid-binding loop between the two binding sites strengthens the toxin interaction with membrane lipids through hydrophobic interactions. BoNT/DC only recognizes sialic acids but not the carbohydrates of gangliosides, thus allowing the interaction with a broad range of acid sialic-containing molecules. The specificity and robust binding of BoNT/DC to target neuron membranes are further achieved by the loop–membrane interaction and by binding to Syt [80]. It is noteworthy that the lipid-binding loop is particularly developed in BoNT/B, DC, and G, which share Syt as protein receptor [79]. A lipid-binding loop is also present in BoNT/C, D, and TeNT, which exhibit two carbohydrate-/ganglioside-binding sites having a main role in toxin-neuronal membrane interaction [110,111,112,113]. In contrast, the lipid-binding loop is not present or is weakly developed in BoNT/A, E, and F, which interact with glycosylated SV2 proteins, supporting that binding to protein-linked glycan ensures sufficient toxin–membrane interaction without requirement of an additional lipid-binding structure.

BoNT/A entry into neuronal cells has been further investigated. In addition to gangliosides and glycosylated SV2, BoNT/A requires Syt-I for endocytosis into synaptic vesicles, despite no BoNT/A direct binding to Syt-I. Using rat primary neuronal cells and derivative cells with gene knockdown by CRISPR (lentiviral clustered regularly interspaced short palindromic repeats) technology, as well as live-cell super-resolution imaging, BoNT/A binds to ganglioside and SV2A, forming nanoclusters on the plasma membrane with restricted lateral mobility, whereas SV2A alone is mobile. Albeit BoNT/A binds to ganglioside or SV2 alone on plasma membrane, the toxin is not engaged in endocytosis. Both gangliosides and SV2 are required for binding to plasma membrane and subsequent endocytosis into synaptic vesicles. Syt-I co-clusters with BoNT/A-ganglioside-SV2 and Syt-I knockdown prevents BoNT/A endocytosis and its further neurotransmission blockade. Thereby, BoNT/A binds concomitantly to the preassembled ganglioside (GT1b, GD1a)-Syt-I complex and to SV2 on the plasma membrane, promoting further interaction between the cytoplasmic domains of Syt-I and SV2. Indeed, BoNT/A binding to SV2 and ganglioside linked to Syt-I brings Syt-I closer to SV2, thus facilitating their interaction (Figure 5). The ganglioside/SV2/Syt-I machinery then triggers BoNT/A uptake into synaptic vesicles. SV2A interaction with Syt-I, through its cytoplasmic domains, controls SV2A-Syt-I nanoclustering at the plasma membrane prior to recruitment in synaptic vesicles and endocytosis. SV2A interaction with the C2 cytoplasmic domains of Syt-I, which are Ca^++^-binding domains, is regulated by Ca^++^ and SV2A phosphorylation. Thus, SV2A, which also interplays with other partners such as adaptor protein 2 and stoning-2, regulates synaptic vesicle recycling, but its exact mode of action remains to be defined. The same mechanism is likely involved in the entry into neuronal cells of BoNT/E and F which use SV2 as a receptor [114,115,116]. It is tempting to speculate that SV2 might also play a role in the neuronal uptake of BoNTs, which recognize Syt and/or gangliosides as receptors. Thereby, BoNT/D, for which gangliosides are the essential receptors, colocalizes with SV2 at the plasma membrane of neuronal cells. Albeit SV2 is required for BoNT/D entry into neuronal cells, no direct protein–protein binding between BoNT/D and SV2 has been shown, indicating that SV2 is not a sensu stricto receptor for BoNT/D but rather a factor involved in its internalization process [117]. Clostridial neurotoxins likely share a general mechanism of internalization into synaptic vesicles based on the ganglioside/SV2/Syt machinery. It is noteworthy that clostridial neurotoxins enter not only recycling synaptic vesicles, but also a pool of non-recyclable vesicles, which can be carriers for retrograde transport, as shown for BoNT/A [118] (see below).

**Figure 6:**
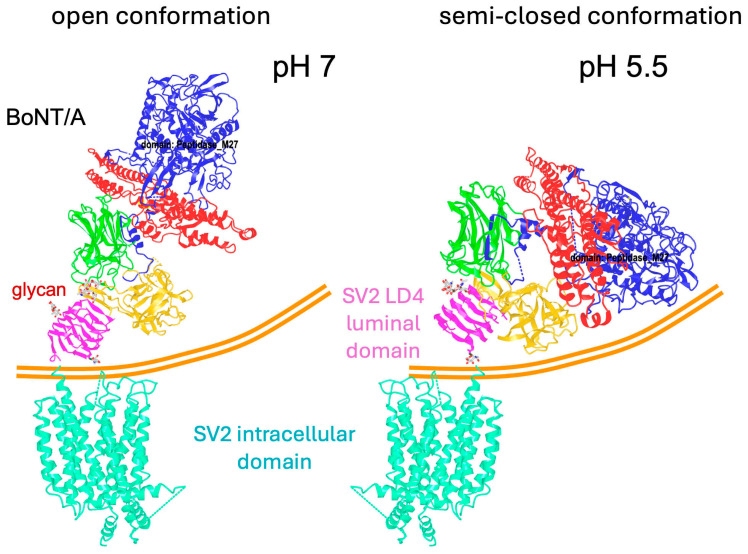
SV2-bound BoNT/A conformation is pH-dependent. At neutral pH, BoNT/A bound to the SV2 luminal domain shows an open conformation, while at pH 5.5 corresponding to acidified endocytic vesicles, BoNT/A moves to a semi-closed conformation facilitating the interaction of HCN and LC with the vesicular membrane and subsequent translocation of LC into the neuronal cytosol. pdb 9F2B, 9F2Y, and 8UO9. The figures were produced with the program CN3D.

Neurotoxin interaction with SV2 was further investigated with BoNT/A. At neutral pH such as in the extracellular space, BoNT/A bound to the SV2 luminal domain retains an open conformation that prevents any contact between LC and HCN with neuronal cell membrane, while at the acidic pH (5.5) of acidified endocytic vesicles, BoNT/A adopts a semi-closed conformation that triggers the interaction of HCN and LC with the vesicular membrane (Figure 6). The BoNT semi-closed conformation facilitates the insertion of the HCN helices into the vesicular membrane which act as chaperone, and subsequent translocation of LC into the cytosol. The closed conformation of BoNT/E which is similar to that of TeNT (Figure 1) likely accounts for the fast entry into neuronal cells and fast onset of symptoms [119]. TeNT also adopts an open conformation at neutral pH similarly to the extended BoNT/A structure, ad a closed conformation at acidic pH (<5.5) [120].

## 5. Intraneuronal BoNT Activity, BoNT/A Versus BoNT/E

LCs are zinc-dependent metalloproteases. LC of each BoNT type and TeNT cleaves specifically one of the three SNARE (soluble N-ethylmaleimide-sensitive-factor attachment receptor) proteins, which include synaptobrevin (or VAMP for vesicle-associated membrane protein), SNAP-25 (synaptosomal-associated protein), and syntaxin, thus leading to the blockade of the neuroexocytosis machinery [1,121,122]. BoNT/A and BoNT/E cleave the same protein substrate, SNAP-25, but at different sites. BoNT/A cleaves at Gln197-Arg198 and BoNT/E at Arg180-Ile181; thus, BoNT/A releases nine C-terminal amino acids and BoNT/E twenty-six residues. The two BoNT types greatly differ in the duration of activity. BoNT/A has a long-lasting effect in neuronal cells, whereas BoNT/E induces a short inhibition of neuroexocytosis. LC/A localizes at the cytosolic side of the membrane, while LC/E remains cytosolic and is degraded by the ubiquitin-proteasome system. In contrast, LC/A is resistant to this degradation pathway. LC/A recruits deubiquitinases, which specifically remove polyubiquitin chains and thus prevent LC/A from proteolyze, through the ubiquitin-proteasome pathway [2,123,124]. A C-terminal di-leucine motif was proposed to mediate LC/A localization at the membrane [125], but an N-terminal region (amino acids 1–17) and an α-helical rich domain (amino acids 268–357, termed low homology domain, LHD) contribute to the plasma membrane localization of LC/A. LHD consists of three regions—an α-helix (275–300) termed R1, loop-α-helix-loop (302–334, R2), and α-helix-loop-α-helix (335–357, R3). R1–R2 play the main role in LC/A localization at the plasma membrane and R2 contributes to SNAP-25 binding [126,127]. Fast synaptic vesicle cycling seems to mediate the LC/A1 intracellular trafficking to the plasma membrane [128].

In addition, SNAP-25 cleavage by LC/A generates the SNAP-25 fragment 1–197, which acts as a dominant negative factor of neuroexocytosis. Indeed, cleavage of a small fraction (10–15%) of SNAP-25 by BoNT/A at the neuromuscular junction is sufficient to induce paralysis [2]. SNAP-25_1–197_ forms a stable and inactive SNARE complex. Moreover, SNAP-25_1–197_ is more stable and has a long half-life in neuronal cells, longer than that of full-length SNAP-25 or LC/A [129,130]. SNAP-25_1–197_ represents an efficient substrate for phosphorylation by the protein kinase C (PKC); thus, SNAP-25_1–197_ is highly phosphorylated at Ser187. Ser187-phosphorylated SNAP-25_1–197_ associates with the plasma membrane with an increased affinity to syntaxin-1A. The exact mode of the dominant negative role of Ser187-phosphorylated SNAP-25_1–197_ remains to be defined. Ser187 phosphorylation might interfere with protein–protein interaction. SNAP-25_1–180_ resulting from BoNT/E cleavage does not contain Ser187 [130]. SNAP-25 cleaved by BoNT/E loses the binding to syntaxin and dissociates from the membrane. Syntaxin is the main player of SNAP-25 retention at the plasma membrane [131]. Thus, BoNT/E cleavage of SNAP-25 destabilizes the four-helical bundle of SNARE complex preventing its binding to membrane and subsequently blocks the neuroexocytosis [132].

## 6. Neuronal Cell Receptor(s) of Tetanus Neurotoxin and Retrograde Transport of TeNT and BoNT

While TeNT enters motor neurons like BoNTs, TeNT uses a different trafficking pathway, including non-acidified endocytic vesicles which undergo an axonal retrograde transport to CNS. Once in the CNS, TeNT enters inhibitory interneurons via an acidic route, allowing the LC translocation into the cytosol and subsequent cleavage of VAMP and blockade of the release of glycine and GABA. TeNT can also use cortical, sensory, and adrenergic neurons for its routing to CNS [122,133]. However, TeNT transits mostly (~80%) through the neuromuscular junction and motor neurons [134]. Interestingly, BoNT/B is the most related BoNT to TeNT at the amino acid sequence level, and both toxins cleave the same intracellular target, VAMP, at the same cleavage site Gln76-Phe77. However, TeNT and BoNT/B inhibit neurotransmitter release in distinct neuronal cells, central inhibitory interneurons, and peripheral cholinergic endings, respectively, and recognize distinct cell surface receptors. Thus, TeNT-dependent blockade of the inhibitory signaling leads to an imbalance between inhibitory and excitatory afferents at the neuromuscular junction. The deficient coordination of muscle contraction results in spastic paralysis [133]. It is noteworthy that in local and cephalic tetanus, TeNT might be produced in high amount in the contaminated wound. Thus, a TeNT fraction might enter neuromuscular junctions via the acidic pathway and cleave locally VAMP leading to an initial and transient flaccid paralysis, which is rapidly overcome by the spastic paralysis due to TeNT activity on the CNS [135,136].

As mentioned above, TeNT contains two ganglioside-binding sites in the HCC_C_ subdomain. The terminal binding site with Trp1289 (W site) as key residue is involved in binding to lactose, which is a sugar component of ganglioside, and the binding site centered on Arg1226 (R site) interacts with sialic acid. Simultaneous binding to two ganglioside molecules is required for high TeNT affinity binding to neuronal membrane and full activity. Gangliosides of “b” series such as GT1b, GD1b bind tightly to the R site, and “a” series gangliosides (GM1a, GD1a) have a higher affinity for the W site. GD2, GD3, GT3, and GT2 can bind also to the R site, and GT1b to the W site [78,137]. However, pretreatment of rat neuronal membranes with protease reduces TeNT binding, indicating that the high affinity TeNT receptor contains a protein part [77].

TeNT binds to nidogen-1 and nidogen-2, which are glycoproteins of the extracellular matrix. The extracellular matrix (also called basal or basement membrane), which overlaps many cells and organs, such as muscle fibers, consists of the following two layers: a felt-like basal lamina in contact with the plasma membrane, and an external fibrillar and reticular lamina, the main components of which are collagen and the glycoprotein laminin. The synaptic cleft of neuromuscular junction is filled with the basal lamina containing collagens, fibronectins, laminins, nidogen, and perlecan as main components [138,139]. Nidogens (nidogen-1 and nidogen-2 in vertebrates) are glycoproteins with three globular domains which bind to a wide range of partners such as laminin, collagen, perlecan, and fibulin. They play a key role as adapter proteins in the organization of the extracellular matrix. Nidogens stabilize the laminin/collagen networks and are notably involved in the development and survival of peripheral nerves and muscles [140,141,142]. Nidogen-2 is particularly enriched in the basal lamina of neuromuscular junctions. It is supported that TeNT interacts with the N1 domain of nidogen through its R site. Since the R site is also involved in ganglioside binding, it is not yet clear whether nidogen and gangliosides bind to the R site simultaneously or sequentially. TeNT bound to nidogen interacts with the cell surface LAR (leucocyte common antigen-related protein) and PTPRδ (protein tyrosine phosphatase receptor δ) of the receptor-type protein tyrosine phosphatases (RPTP) family, which belongs to the transmembrane receptor-like protein tyrosine phosphatases. Thus, LAR and PTPRδ are transmembrane proteins which act as neuronal receptors for TeNT [143]. Moreover, LAR and PTPRδ contribute to the formation and organization of synapses [144]. Phosphatase activity of LAR and PTPRδ regulates signaling molecules such as phosphorylation of TrkB (tropomyosin receptor kinase B or tyrosine receptor kinase B) and BDNF (brain-derived neurotrophic factor). However, TeNT-nidogen binding to LAR is independent of LAR phosphatase activity [143]. The TeNT-nidogen-gangliosides complex is driven through LAR and PTPRδ in the retrograde axonal route containing the TrkB and p75^NTR^ (P75 neurotrophic receptor) neurotrophin receptors and thus avoids the synaptic vesicle recycling with entry into acidified vesicles [145,146]. TeNT binds to gangliosides in membrane lipid microdomains and enters motor neurons via a specialized clathrin pathway, which is independent from the synaptic vesicle recycling and dependent on dynamin and the accessory proteins AP-2 and AP180 [147]. LAR is also localized in membrane lipid microdomains with caveolin, likely with TeNT [148,149]. Caveolin is an essential component of caveolae, which are endosomes with neutral pH distinct from clathrin endosomes and which are mainly involved in the internalization of membrane components enriched in lipid microdomains [150,151]. The intracellular compartment used for TeNT retrograde transport remains to be refined. TeNT enters tubules and bright vesicles which share a similar bright appearance with caveolae. However, TeNT uptake requires the adaptor proteins of the clathrin pathway, AP-2 and AP180, but not epsin1, indicating that TeNT might use a particular clathrin pathway. Interestingly, the ganglioside GD1b associates with TeNT at the neuronal surface and it is no longer detected in the TeNT-containing intracellular compartment [147,152]. Gangliosides seem to only be required for the specific TeNT binding at the neuronal surface, and not for the TeNT intracellular transport. RPTPs have multiple functions, including the recruitment of synaptic vesicles to the active zone and endosome signaling to the retrograde transport machinery [153]. Thus, TeNT gains access to Rab5 early endosomes. Then, Rab5 is exchanged with Rab7, a marker of late endosome, which is required for fast retrograde axonal transport [154]. Thereby, TeNT uses the neurotrophin microtubule-based transport in nonacidic vesicles containing TrkB and p75^NTR^ and the motor molecules, dynein and kinesin [133,145,155]. In the central nervous system, the whole TeNT is delivered to the extracellular space and enters the final target neurons, which are inhibitory interneurons involved in the regulation of motor neuron activity.

**Figure 7:**
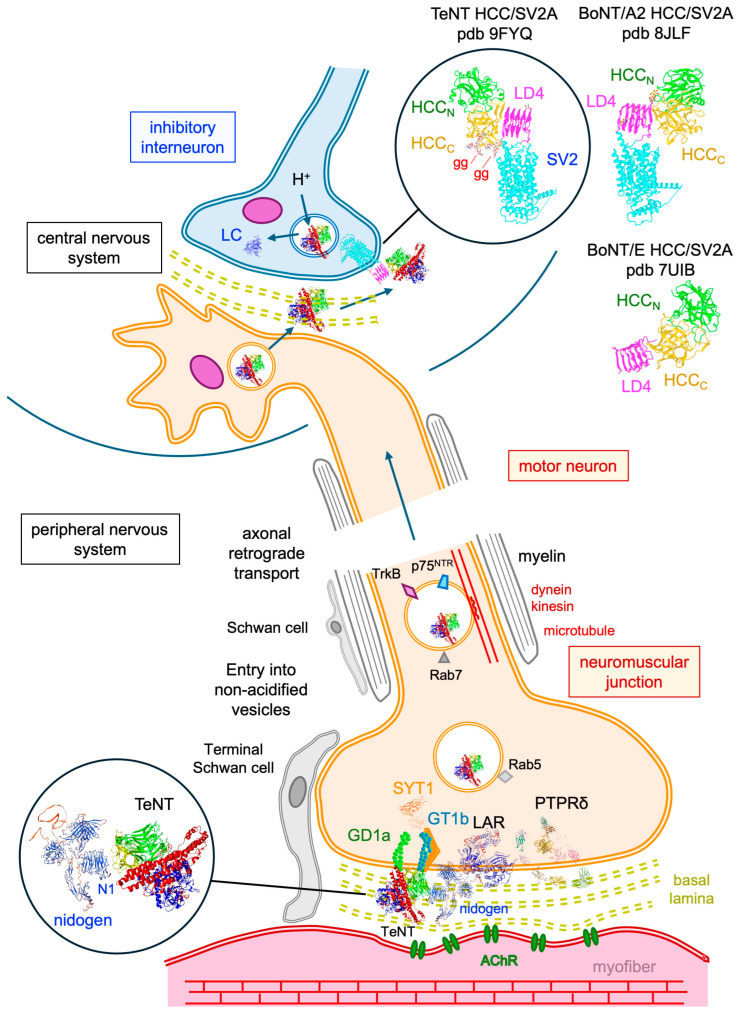
Tetanus neurotoxin (TeNT) receptors and trafficking from the motor neuron to central inhibitory interneuron. TeNT binds to nidogen (UniProt P14543) in the extracellular matrix of neuromuscular junction and subsequently to gangliosides, LAR (UniProt P10586), and PTPRδ (UniProt P23468) as specific receptors on neuronal cell surface. The toxin is driven into a neutral vesicular pathway which retrogradely transports neurotrophic factors to the cell body in the central nervous system. The axonal retrograde pathway uses signaling molecules such as the neurotrophin receptors TrkB and p75^NTR^, Rab7, and motor molecules (dynein, kinesin). Whole and intact TeNT is transcytosed in the intercellular space of the central nervous system. TeNT then enters inhibitory interneurons through specific interaction with gangliosides and SV2A or SV2B. TeNT HCC_C_ binds to the N-terminal β-strand of the luminal domain (LD4) of SV2A, while BoNT/A and BoNT/E interact with the glycosylated C-terminal β-strand of the LD4 β-helix. SV2 binding site is localized in BoNT/E HCC_C_ and at the interface of HCC_N_ and HCC_C_ in BoNT/A. In the target inhibitory interneurons, TeNT is routed into an acidic vesicular pathway which mediates the translocation of LC into the cytosol and subsequent cleavage of the intracellular target, VAMP, leading to the blockade of neurotransmitter release. Terminal Schwann cells have a trophic function of axons and modulate neuromuscular transmission, and Schwann cells along the axons produce myelin [191]. The figures were produced with the CN3D program. gg, ganglioside; AChR, acetylcholine receptor.

TeNT entry into central interneurons is ganglioside- and SV2-dependent. Indeed, TeNT binds to SV2A and SV2B, but not to SV2C. TeNT interaction with SV2A is different from that of BoNT/A and BoNT/E with SV2. SV2A binding site is located in the center of TeNT HCC_C_ (Ser1156-Arg1168) and interacts with the N-terminal part of the SV2A LD4 domain. The TeNT segment Ser1156-Arg1168 forms a β-hairpin which interacts with the N-terminal β-strand of SV2 LD4 β-helix, while BoNT/A and BoNT/E bind to glycosylated LD4 C-terminal β-strand (Figure 7). Similarly to TeNT, the SV2 binding site is located in the HCC_C_ center of BoNT/E, while BoNT/A uses the interface between HCC_N_ and HCC_C_ to interact with SV2. Both SV2 LD4 protein part and N-glycan are recognized by the BoNT/A interface between the two HCC subdomains. These structural investigations corroborate the observations that BoNT/A HCC_N_ contributes to high-affinity binding to neuronal receptor [156] and that both HCC_N_ and HCC_C_ subdomains are required to induce neutralizing antibodies against BoNT/A [157]. BoNT/E shows a different mode of interaction with SV2. In addition to protein–protein interaction between HCC_C_ and SV2 LD4 C-terminal β-strands, BoNT/E recognizes the glycan part by a distinct site in HCC_N_. Indeed, Asn573 glycan extends towards a hydrophobic pocket in the HCC_N_ domain. In contrast to BoNT/A and BoNT/E, TeNT binding to SV2A or SV2B requires no glycosylation of SV2. However, TeNT interacts with sugars of gangliosides bound to R and W sites, which are close to the SV2 binding site. As discussed above, Syts and gangliosides are interconnected, as well as SV2s and Syts, through their cytoplasmic domains. Thereby, the SV2s-Syts-gangliosides complex constitutes specific and high-affinity receptors for clostridial neurotoxins on target neuronal cells [85,87,94,158,159,160] (Figure 7). SV2A is widespread in the central nervous system, notably in the brain, and only in a subset of motor neurons, whereas SV2B and to a lower extent SV2C are localized in restricted areas of the brain. In contrast, the isoforms SV2C and SV2B, which are the targets of BoNT/A and BoNT/E, are expressed in all motor neurons, and SV2A is confined to only slow motor neurons [82,161]. The preferential targeting of SV2A by TeNT supports its main activity in the central nervous system. The extracellular matrix of the central nervous system forming the perineuronal nets is characterized by the predominance of nonfibrous components such as glycoseaminoglycans (hyaluronic acid, heparan sulfate, and chondroitin) and proteoglycans such as perlecan, nidogen, and low amounts of fibrous glycoproteins (laminin, fibronectin, and tenascins) [162,163]. It is not yet defined whether TeNT uses nidogen in the extracellular matrix of the perineural net to enter the inhibitory interneurons.

TeNT enters inhibitory interneurons via acidified vesicles leading to the translocation of the LC chain into the cytosol. LC delivery into the cytosol is achieved by reduction of the disulfide bond between LC and HC with the cellular thioredoxin–thioredoxin reductase system [164,165]. Subsequently, TeNT LC cleaves VAMP. Thus, TeNT uses SV2 and gangliosides for entry into the vesicular acidic pathway of central interneurons.

BoNTs have also been shown to undergo a retrograde axonal trafficking until the central nervous system, albeit to a lower efficiency compared to TeNT. Using fluorescent full-length BoNT/A and BoNT/E, as well as their corresponding HC domains in cultured motor neurons, Schiavo et al. showed that a fraction of BoNT enters motor neurons via non-acidified compartments and undergo axonal retrograde transport. Thus, BoNT HC domains like TeNT HC retain the addressing sites for the sorting in the retrograde transport pathway. Colocalization of BoNT/A or BoNT/E HC with TeNT HC indicates that BoNT uses similar transport vesicles labeled with p75^NTR^. Interestingly, the speed of BoNT retrograde transport is similar to that of TeNT (average speed 0.8 µm/s) [166]. BoNT/A has been found to bind with high affinity to the fibroblast growth factor receptor 3 (FGFR3), which is expressed in motor neuron terminals and to induce FGFR3 dimerization and uptake in neuronal cells under resting and stimulated conditions [167,168]. BoNT/A might exploit the FGFR3 endocytic pathway to enter a non-acidified compartment and undergo the retrograde transport [169]. Alternatively, BoNT/A retrograde transport might be mediated by autophagosomes, which are acidified compartments upregulated by neuronal stimulation [170]. However, the signaling molecules and receptors used for BoNT sorting into the retrograde pathway remain to be further determined.

BoNT retrograde transport has also been demonstrated in in vivo models. BoNT/A activity was monitored by the sensitive detection method of SNAP-25 cleavage. BoNT/A injection into the superior colliculus of rat, a brain area which controls the eye movements and contains neurons connected to retinal neurons, induced cleaved SNAP-25 in the ipsilateral retinal cells at day 1 and in the contralateral retina three days later, albeit to a lower extent. Section of the optic nerve or administration of colchicine, which inhibits tubulin polymerization, prevents the BoNT/A activity in the contralateral retina, supporting a BoNT/A retrograde and transsynaptic transport [171,172]. Moreover, following BoNT/A injection into the limb muscle, cleaved SNAP-25 was detected in the ipsilateral and contralateral spinal cord areas, and paralysis was recorded not only in the ipsilateral but also in the contralateral limb [173,174,175]. This further highlights that BoNT can traffic from neuromuscular junction to CNS, with wider spreading of BoNT/A1 than BoNT/A2 in the spinal cord [174]. Similarly to TeNT, BoNT/A is, at least partially, retrogradely transported to the CNS, after which the full length neurotoxin is released from motor neuron and enters preferentially cholinergic central neurons, whereas TeNT targets inhibitory interneurons [176]. Further evidence of BoNT retrograde and transsynaptic transport was provided by using compartmentalized microfluidic devices where BoNT/A, BoNT/D, as well as TeNT undergo transcytosis and interneuronal transfer [177]. In addition, radiolabeled BoNT/A injected into the rat bladder is detected in the respective lumbosacral spinal cord segment and dorsal root ganglia, indicating a BoNT transport through sympathetic neurons and transfer to sensory neurons, leading to changes in sensory, sympathetic, and cholinergic markers involved in the control of bladder function [178,179]. Numerous clinical observations show that the beneficial therapeutic effects of BoNT/A, which is the BoNT type most used in medical applications, result not only from the toxin activity at the neuromuscular junctions but also from central effects. The following two main therapeutic outcomes support a contribution of BoNT central effects: movement disorder recovery and pain modulation. Dystonia and spasticity are characterized by involuntary and impaired movements, spasms, and abnormal posturing and result from hyperactivity and intermittent contractions of certain muscles associated with disordered sensorimotor control. Intramuscular injection of BoNT/A induces local and distant effects in non-injected muscles, such as motricity reduction in the injected agonist muscle and increased activity of the non-injected antagonist muscle. Alpha motor neurons innervating the neuromuscular junction (in the agonist muscle, for example) are connected to Renshaw cells in the spinal cord which mediate recurrent inhibition of other motor neurons (those of the antagonist muscles, for example). A BoNT/A central effect likely resides in blocking the inhibitory control from Renshaw cells. Thus, it is assumed that BoNT/A can modulate central circuits such as activation or deactivation of somatosensory circuits. BoNT/A-induced changes in SNC organization have been corroborated by electrophysiological studies [169,176,180,181,182,183,184,185]. Modulation of central neuronal circuits by BoNT/A includes also the nociceptive circuits leading to neuropathic pain. BoNT/A is used in the treatment of various chronic neuropathic pains (migraine, amputation, peripheral nerve or spinal cord injury, painful radiculopathy, post-stroke pain, multiple sclerosis-related pain, etc.). BoNT/A antinociceptive effects were investigated in various animal pain models. The central mechanisms of BoNT/A pain relief include inhibition of neurotransmitter release in specific sensory neurons such as TRPV1 (transient receptor potential vanilloid type 1)-expressing neurons, reduction in microglia activation and release of pro-inflammatory cytokines, reduction in neuroinflammation, and modulation or reorganization of nociceptive circuits) [169,186,187,188,189,190]. However, the precise modes of BoNT/A analgesic activity are still lacking.

## 7. Conclusions

Clostridial neurotoxins are among the most potent toxins, including bacterial, animal, plant toxins, and toxic chemical compounds. Their extreme potency does not relate to a hyper-toxic step of activity, but to cumulative effects of successive steps, with each of them displaying a limited effect [3]. The first critical step consists of the toxin trafficking in the host until the target neurons, including BoNT passage through the intestinal barrier and TeNT axonal retrograde, transport to the central nervous system. As discussed, BoNTs associate with non-toxic proteins (L/HA or L/OrfX complexes) which confer resistance to acidic pH and protease degradation in the host digestive tract and facilitate BoNT passage across the intestinal barrier. TeNT which does not form any complex with non-toxic proteins is not stable in the digestive tract and does not induce tetanus from the oral route. In contrast, TeNT efficiently uses motor neurons for its retrograde transport to the central nervous system. Binding to specific receptors promotes clostridial neurotoxin uptake and concentration to target neuronal cells, avoiding dispersion and degradation in non-toxin active compartment of the host. High-affinity receptors of clostridial neurotoxins are achieved by recognition of protein and glycan domains of clustered membrane components, including gangliosides and the synaptic vesicle proteins SV2s and Syts. TeNT and each BoNT type interact with distinct sets of these component domains and through different modes. Synaptic vesicle proteins drive the neurotoxins in an acidified endocytic pathway mediating LC translocation into the cytosol and subsequent cleavage of a clostridial neurotoxin-specific SNARE protein. TeNT exploits an extracellular component (nidogen) and its corresponding membrane partners (LAR, PTPRδ) as sorting machinery into the axonal retrograde transport at neutral pH, prior to target central interneurons in a ganglioside-/SV2A-dependent manner. The extreme potency of clostridial neurotoxins is further achieved by their selective intracellular activity. Albeit clostridial neurotoxins are not super proteolytic enzymes, they cleave critical targets of the neuroexocytosis machinery. Indeed, cleavage of only a small fraction of SNARE protein such as SNAP25 is sufficient to block the neuroexocytose [2,130]. Moreover, only a partial muscle paralysis is sufficient to induce muscle weakness and asphyxia [3].

Better understanding of the mode of action of clostridial neurotoxins paves the way for development of more efficient strategies of prevention and therapy of botulism and tetanus. Not only are clostridial toxins responsible for severe diseases but they are also powerful therapeutic tools, notably BoNTs. Detailed knowledge of their mechanism of action allows the engineering of novel molecules for specific applications such as recombinant BoNTs with prolonged duration of action, with increased activity in humans, targeting sensory neurons for pain treatment or non-neuronal cells, and transport of drug into the central nervous system [192,193,194,195,196,197,198]. For example, the analysis of structural interaction of BoNT/B with human and mouse Syt receptor led to an engineered BoNT/B with improved activity in humans [199]. Among the pathogenicity mechanisms of neurodegenerative diseases, which are still poorly understood, dysregulation of membrane components and subsequent compromised signal transduction, as well as impairment of neurotrophic factor transport, seem to be critical [200,201,202,203]. Clostridial neurotoxins represent appropriate tools to explore organization and coordination of major membrane receptor components and intracellular transport.

## Data Availability

No new data were created or analyzed in this study.

## Abbreviations

The following abbreviations are used in this manuscript

BoNT	Botulinum neurotoxin
TeNT	Tetanus neurotoxin
CNS	Central nervous system
HA	Hemagglutinin
LC	Light chain
HC	Heavy chain
HCN	Heavy chain, N-terminal domain
HCC	Heavy chain, C-terminal domain
HCC_N_	HCC, N-subterminal domain
HCC_C_	HCC, C-subterminal domain
NTNH	Non-toxic non-hemagglutinin
LD4	Luminal domain 4
SNARE	Soluble N-ethylmaleimide-sensitive-factor attachment receptor
SNAP	Synaptosomal-associated protein
SV2	Synaptic vesicle protein 2
SYT	Synaptotagmin
LAR	Leucocyte common antigen-related protein
PTPRδ	Protein tyrosine phosphatase receptor δ
VAMP	Vesicle-associated membrane protein
